# Minimally Invasive Treatment for Lumbar Disc Herniation: A Matched Comparison Between Tubular Microdiscectomy and Percutaneous Endoscopic Lumbar Discectomy

**DOI:** 10.7759/cureus.57589

**Published:** 2024-04-04

**Authors:** Pedro Teles, Paulo Pereira, Carolina Silva, Rui Vaz, Pedro Santos Silva

**Affiliations:** 1 Department of Neurosurgery, Centro Hospitalar Universitário de São João, Porto, PRT; 2 Faculty of Medicine, University of Porto, Porto, PRT

**Keywords:** degenerative spine disease, spine microsurgery, tubular microdiscectomy, endoscopy, minimally invasive

## Abstract

Background and objectives

Discectomy for lumbar disc herniation is the most common spinal surgical procedure. Technological advances have led to the emergence of minimally invasive surgical approaches such as tubular microdiscectomy (TMD) and percutaneous endoscopic lumbar discectomy (PELD). The purpose of this study was to compare the clinical outcomes of PELD to those of TMD at one-year follow-up.

Materials and methods

This observational registry-based (Spine Tango) cohort study included patients with symptomatic lumbar disc herniation submitted to PELD or TMD. The inclusion criteria were patients who underwent minimally invasive lumbar discectomy (PELD or TMD), patients who attended a follow-up after a minimum of 12 months post surgery, and valid pre- and postoperative questionaries. The primary endpoint was defined as the difference between pre- and postoperative Core Outcome Measures Index (COMI) for the back. The matching was based on a 1:1 nearest neighbor matching without replacement.

Results

A total of 109 patients were included in this study. Propensity score matching (PSM) was performed achieving 86 patients in the matched sample. Regarding COMI improvement, we found no significant difference between the PELD and TMD groups (paired t-test: estimate, -0.23; standard error, 0.6; p=0.7), and we also did not find any significant difference between groups concerning Oswestry Disability Index (ODI) and EuroQol 5 Dimension (EQ-5D). Medication usage and return to work were similar among the matched groups.

Conclusions

PELD is a technique that minimizes tissue damage achieving good clinical outcomes similar to TMD. This was observed one year after surgery from patient-reported outcome measures (PROMs) that measured pain improvement, disability, and quality of life.

## Introduction

Lumbar disc herniation has a lifetime incidence between 13% and 40% and is more common among the working population, hence its high social and economic impact. It can be self-limited, and most patients can recover with conservative therapies [1,2]. Surgical treatment is reserved for conservative management failures and for patients who develop neurological deficits [1,2].

Discectomy is the most effective surgical treatment for a symptomatic patient with a herniated lumbar disc. Technological advances including microscopic magnification and tubular retractors have led to the emergence of tubular microdiscectomy (TMD), which can translate into shorter hospital stays, lower blood loss and postoperative analgesic usage, and earlier return to daily life or work [3]. More recently, percutaneous endoscopic lumbar discectomy (PELD) has become popular, with the same advantages that tubular surgery has shown over open discectomy, claiming lesser surgical risks and fewer social burdens [4]. Minimally invasive approaches, such as PELD, are frequently compared to open discectomy in clinical studies. However, there is a limited number of studies that have directly compared the outcomes of PELD to TMD. Considering the learning curve of endoscopic surgery, understanding whether transitioning from TMD to endoscopy can yield equivalent clinical outcomes is crucial. As the demand for minimally invasive procedures continues to rise, it becomes important to evaluate and compare the outcomes of these two techniques to optimize patient care and inform clinical decision-making.

The purpose of this study is to compare the clinical outcomes of PELD to those of TMD at one-year follow-up using a propensity score matching (PSM) analysis.

## Materials and methods

Study design and participants

This observational retrospective registry-based (EuroSpine Spine Tango) cohort study included patients diagnosed with symptomatic lumbar disc herniation submitted to PELD or TMD in the spine unit of a neurosurgical department of a Portuguese university hospital, from January 2019 to May 2021, with a 12-month follow-up. The TMD group included patients treated before the PELD became a standardized technique at our center.

Comparative observational studies can be affected by the inherent variability in patient characteristics and preoperative conditions. To address potential confounding factors and enhance the robustness of comparative analysis, propensity score matching (PSM) allows for the creation of balanced groups by matching individuals based on a set of predetermined variables. By minimizing selection bias and accounting for baseline differences, PSM enables a more rigorous comparison of outcomes between two surgical techniques, enhancing the internal validity of a study.

Patients gave consent to be enrolled in the registry, and Strengthening the Reporting of Observational Studies in Epidemiology (STROBE) guidelines were followed for writing.

The inclusion criteria were patients who underwent minimally invasive lumbar discectomy (PELD or TMD), patients who attended a follow-up after a minimum of 12 months post surgery, and valid pre- and postoperative questionaries. The exclusion criteria included age under 18 years, non-degenerative spine pathology (such as infection, trauma, or tumor), need for central decompression or foraminoplasty, more than one level of surgery, and previous spine fusion.

Surgical technique and postoperative recommendations

The operative procedures are standardized in our center, and all surgeries were performed by four surgeons experienced in minimally invasive techniques. TMD were performed with METRx (Medtronic Minimally Invasive Therapies, Minneapolis, MN) tubular retractor system, using 18 mm-diameter tubes, in accordance with the techniques described earlier [3,5]. PELD were performed with iLESSYS and TESSYS (Joimax, Karlsruhe, Germany) for interlaminar (IL) and transforaminal (TF) procedures, respectively, in accordance with previously described techniques [6].

Patients in both groups were instructed to restrict intense physical activity for four weeks, but no postoperative instruction preventing return to work took place at the moment of discharge. Patients were encouraged to return to work as soon as they felt confident to do so.

Clinical data

Clinical and demographic data was collected from clinical records including age, gender, body mass index (BMI), current smoker status, occupation, depression/anxiety history, previous spine surgery, American Society of Anesthesiologists (ASA) classification, symptom duration, discectomy technique, level of disc herniation, disc herniation type (extrusion and protrusion), disc herniation location (posterolateral and foraminal/extraforaminal), and complications.

The Spine Tango registry was accessed to collect the patient-reported outcome measures (PROMs), before and one year after surgery, including the Core Outcome Measures Index (COMI) for the back, EuroQoL 5 Dimension (EQ-5D) questionary, and Oswestry Disability Index (ODI). These PROMs were self-completed by the patients.

Outcomes

The primary endpoint of the study was defined as the difference between pre- and postoperative COMI including pain severity and the impact of the pain in daily activities. Additionally, return to work and postoperative medication usage were accessed at one year postoperatively. The clinical outcome defined by the surgeon was done according to the Odom criteria.

Matching, sample size calculation, and statistical analysis

Sample size calculation was based on an effect size of 0.6 (based on Portuguese COMI minimal detectable change of 1.7 and an SD of 2.8) [7,8]. The sample size was calculated as 40 patients for a two-sided paired t-test (with an alpha of 0.05 and a power of 0.95). The enrolment aimed for 100 patients to account for losses related to matching.

R software (R Foundation for Statistical Computing, Vienna, Austria) version 4.2.1 was used for data analysis.

The matching was based on a 1:1 nearest neighbor matching without replacement. Propensity score was used as distance and estimated with logistic regression, targeting the average treatment effect. The following covariates were used for matching: disc level, hernia location, gender, smoker status, depression/anxiety history, previous spine surgery symptom duration, age (<40, 41-65, and >65), BMI (split at 30), and preoperative COMI (split at 7, based on the first quartile). Covariate assessment was based on standardized mean difference.

Effect estimation after matching was done using a two-sided paired t-test without covariate adjustment; cluster-robust standard errors were estimated based on pair membership.

The comparison of continuous variables between groups was performed with the Kruskal-Wallis test.

## Results

**Figure 1:**
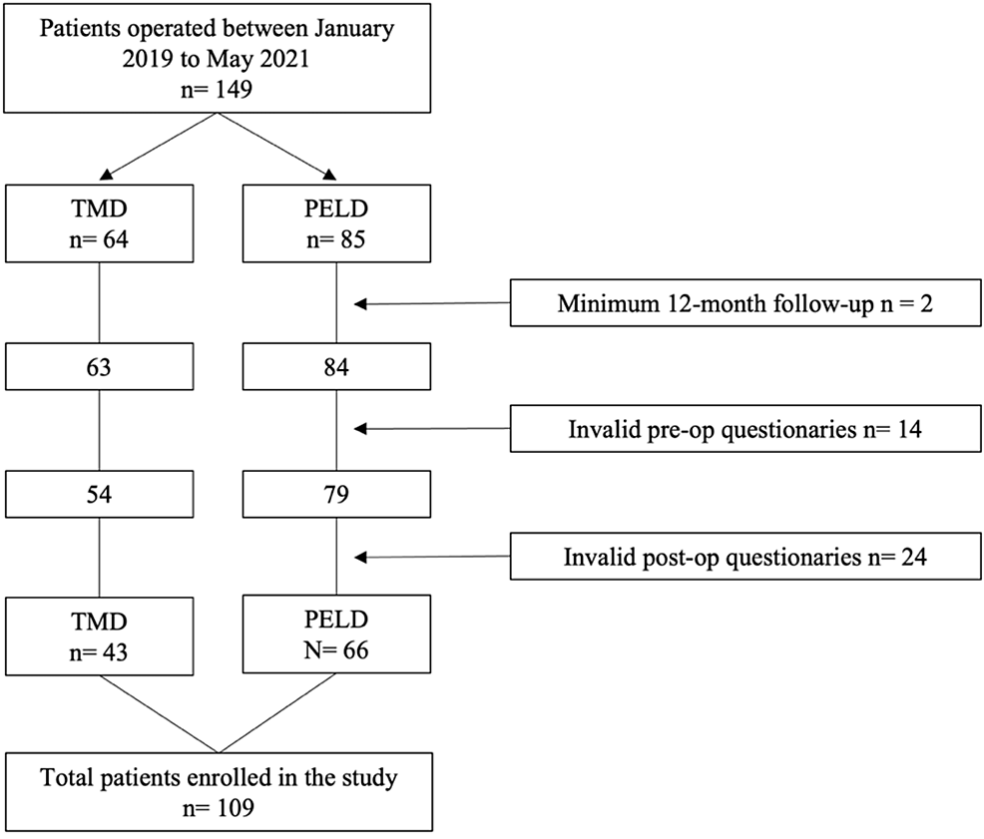
Flowchart of patient selection TMD, tubular microdiscectomy; PELD, percutaneous endoscopic lumbar discectomy

A total of 109 patients were included in this study, out of which 66 were submitted to PELD and 43 to TMD (Figure 1). The mean age was 46.3 (SD: 12.5), and 65 patients (59.6%) were females. The symptom duration was more than 12 months in 32 patients (29.4%), and 11 patients (10.1%) had previous spine surgery. The location of the herniation was foraminal or extraforaminal in 13 cases (11.9%), and 58 patients (53.2%) underwent surgery at the L5-S1 level. The interlaminar approach was used in 42 patients submitted to PELD (63.6%).

Five patients (4.6%) presented a surgery-related complication, three of which occurred in the PELD group and two in the TMD group. The nature of these complications was transitory (subcutaneous infection, subcutaneous hematoma, sensory dysfunction, and early recurrence), except for one case submitted to transforaminal PELD, with persistent L4-sensitive radiculopathy. We also registered four reoperations for disc herniation recurrence, two in each group.

**Figure 2:**
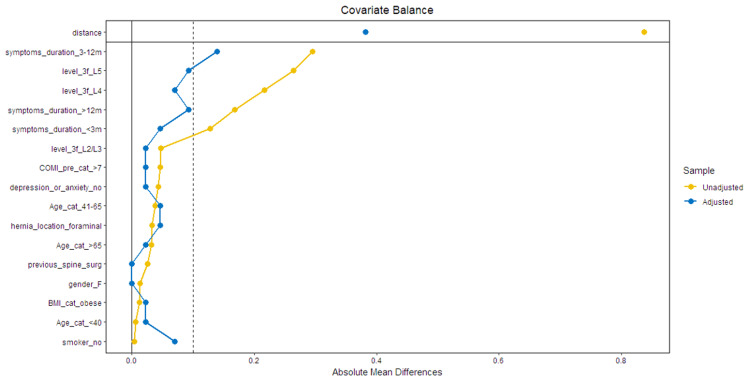
Love plot for covariate balance assessment after matching The dashed line representing the desired target of 0.1 for the standardized mean difference COMI, Core Outcome Measures Index; BMI, body mass index

Propensity score matching was performed, achieving 86 patients in the matched sample. Patient characteristics are summarized in Table 1. Covariate balance before and after matching is illustrated in Figure 2.

**Table 1:** Frequency of the overall patient characteristics BMI, body mass index; Q1, first quartile; Q3, third quartile; ASA, American Society of Anesthesiologists; PELD, percutaneous endoscopic lumbar discectomy; TMD, tubular microdiscectomy

	PELD (N=43)	TMD (N=43)	Total (N=86)
Age			
Median (Q1 and Q3)	46.00 (40.50 and 54.00)	45.00 (37.50 and 56.00)	45.00 (39.25 and 55.00)
Gender			
Male	17 (39.5%)	17 (39.5%)	34 (39.5%)
Female	26 (60.5%)	26 (60.5%)	52 (60.5%)
BMI			
Median (Q1 and Q3)	25.78 (22.69 and 29.77)	26.27 (24.05 and 28.31)	26.17 (23.05 and 29.76)
Smoker			
Yes	4 (9.3%)	7 (16.3%)	11 (12.8%)
No	39 (90.7%)	36 (83.7%)	75 (87.2%)
Demanding job			
Yes	26 (60.5%)	21 (48.8%)	47 (54.7%)
No	17 (39.5%)	22 (51.2%)	39 (45.3%)
Depression or anxiety			
Yes	10 (23.3%)	11 (25.6%)	21 (24.4%)
No	33 (76.7%)	32 (74.4%)	65 (75.6%)
Previous spine surgery			
Yes	5 (11.6%)	5 (11.6%)	10 (11.6%)
No	38 (88.4%)	38 (88.4%)	76 (88.4%)
ASA			
1	26 (60.5%)	26 (60.5%)	52 (60.5%)
2	17 (39.5%)	16 (37.2%)	33 (38.4%)
3	0 (0.0%)	1 (2.3%)	1 (1.2%)
Symptom duration			
<3 months	10 (23.3%)	12 (27.9%)	22 (25.6%)
3-12 months	20 (46.5%)	14 (32.6%)	34 (39.5%)
>12 months	13 (30.2%)	17 (39.5%)	30 (34.9%)
Level			
L2-L3	0 (0.0%)	2 (4.7%)	2 (2.3%)
L3-L4	3 (7.0%)	2 (4.7%)	5 (5.8%)
L4-L5	20 (46.5%)	23 (53.5%)	43 (50.0%)
L5-S1	20 (46.5%)	16 (37.2%)	36 (41.9%)
Hernia location			
Posterolateral	39 (90.7%)	37 (86.0%)	76 (88.4%)
Foraminal	4 (9.3%)	6 (14.0%)	10 (11.6%)
Herniation type			
Protrusion	19 (44.2%)	25 (58.1%)	44 (51.2%)
Extrusion	24 (55.8%)	18 (41.9%)	42 (48.8%)
Return to work			
Yes	34 (85.0%)	31 (86.1%)	65 (85.5%)
No	6 (15.0%)	5 (13.9%)	11 (14.5%)
Return to work (weeks)			
Median (Q1 and Q3)	5.50 (4.00 and 8.50)	5.50 (4.00 and 12.00)	5.50 (4.00 and 11.50)
Postoperative analgesic medication			
WHO I	15 (34.9%)	13 (31.0%)	28 (32.9%)
WHO II	0 (0.0%)	4 (9.5%)	4 (4.7%)
WHO III	2 (4.7%)	1 (2.4%)	3 (3.5%)
None	26 (60.5%)	24 (57.1%)	50 (58.8%)
Medication duration (weeks)			
Median (Q1 and Q3)	0.00 (0.00 and 1.00)	0.40 (0.00 and 10.00)	0.00 (0.00 and 8.00)
Odom			
Excellent	20 (47.6%)	22 (51.2%)	42 (49.4%)
Good	14 (33.3%)	14 (32.6%)	28 (32.9%)
Fair	3 (7.1%)	6 (14.0%)	9 (10.6%)
Poor	5 (11.9%)	1 (2.3%)	6 (7.1%)

**Figure 3:**
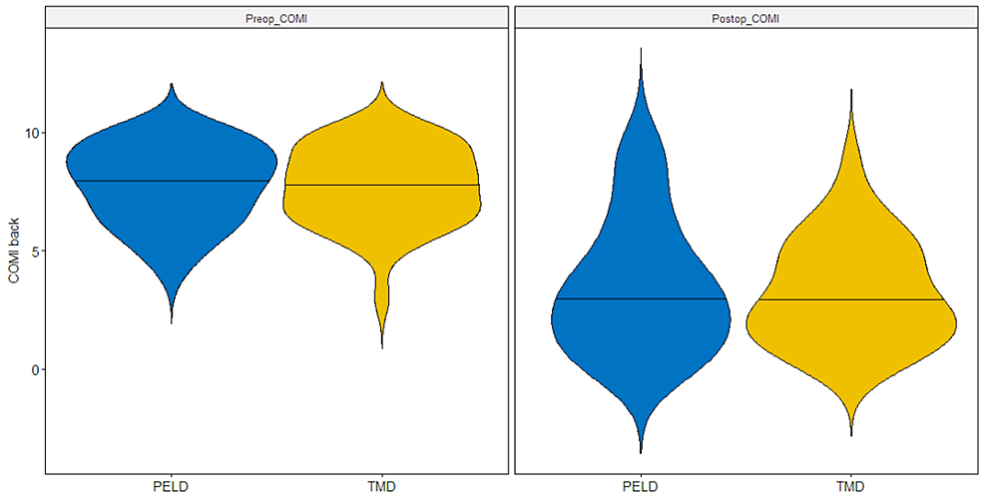
Violin plot showing the distribution of COMI for the back score in pre- and postoperative periods, in the PELD and TMD groups, after PSM COMI, Core Outcome Measures Index; PELD, percutaneous endoscopic lumbar discectomy; TMD, tubular microdiscectomy; PSM, propensity score matching

We performed subgroup analysis in patients submitted to PELD with transforaminal (TF) and interlaminar (IL) technique and found no significant difference in COMI improvement after surgery (median COMI improvement: PELD IL, -5.0; PELD TF, -4.0; TMD, -4.5; Kruskal-Wallis, p=0.92) (Figure 3).

**Figure 4:**
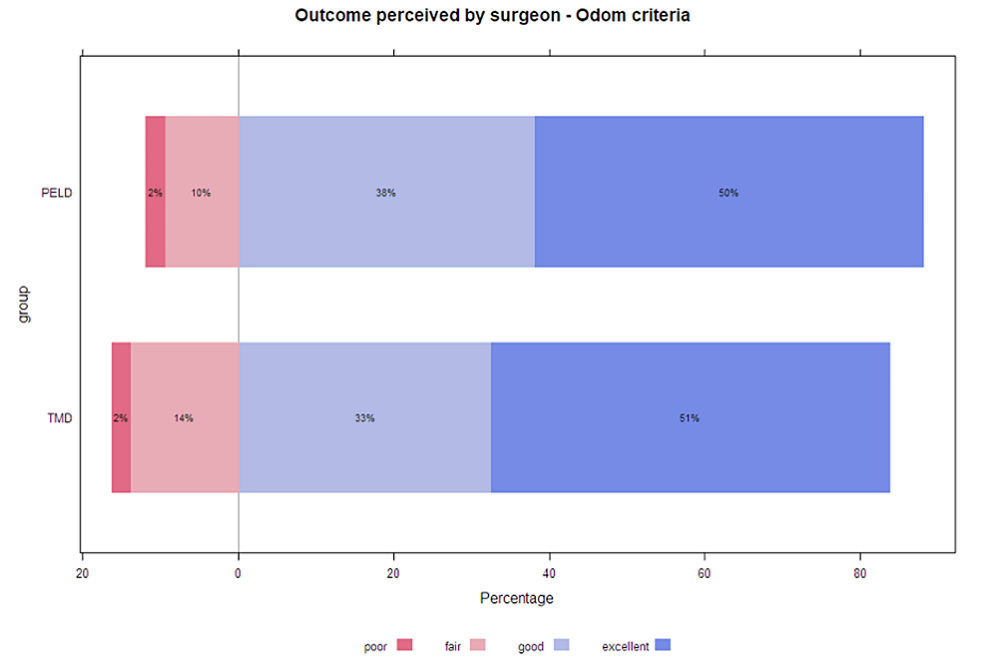
Comparative stacked bar for surgical outcome according to the Odom criteria PELD, percutaneous endoscopic lumbar discectomy; TMD, tubular microdiscectomy

We also did not find any significant difference between groups concerning ODI and EQ-5D. Preoperative, postoperative, and PROM improvement are summarized in Table 2. Return to work and the duration of postoperative analgesic use were similar among the matched groups (paired t-test: p=0.77 and p=0.06, respectively). Additionally, the outcomes according to surgeons were equivalent in both groups (Figure 4).

**Table 2:** Summary of PROMs (preoperative, postoperative, and improvements) PELD, percutaneous endoscopic lumbar discectomy; TMD, tubular microdiscectomy; COMI, Core Outcome Measures Index; NRS, numeric rating scale; ODI, Oswestry Disability Index; EQ-5D, EuroQol 5 Dimension; Q1, first quartile; Q3, third quartile; PROMs, patient-reported outcome measures

	PELD (N=43)	TMD (N=43)	Total (N=86)
Preoperative COMI			
Median (Q1 and Q3)	8.00 (6.85 and 9.00)	8.00 (6.50 and 9.00)	8.00 (6.50 and 9.00)
Preoperative NRS back			
Median (Q1 and Q3)	6.00 (2.75 and 7.20)	6.00 (5.00 and 8.00)	6.00 (4.00 and 8.00)
Preoperative NRS leg			
Median (Q1 and Q3)	8.00 (6.00 and 9.00)	8.00 (6.75 and 9.00)	8.00 (6.00 and 9.00)
Preoperative ODI			
Median (Q1 and Q3)	42.11 (34.50 and 58.00)	54.00 (40.00 and 68.00)	50.00 (37.00 and 65.00)
Preoperative EQ-5D			
Median (Q1 and Q3)	0.52 (0.19 and 0.69)	0.44 (0.03 and 0.61)	0.52 (0.08 and 0.62)
Postoperative COMI			
Median (Q1 and Q3)	3.00 (1.05 and 5.00)	3.00 (1.55 and 5.00)	3.00 (1.20 and 5.00)
Postoperative NRS back			
Median (Q1 and Q3)	3.00 (0.50 and 5.00)	4.00 (1.00 and 5.00)	3.00 (1.00 and 5.00)
Postoperative NRS leg			
Median (Q1 and Q3)	2.00 (0.00 and 4.00)	3.00 (0.00 and 5.00)	2.00 (0.00 and 4.00)
Postoperative ODI			
Median (Q1 and Q3)	12.00 (6.00 and 27.67)	21.000 (7.50 and 36.12)	16.00 (6.00 and 33.00)
Postoperative EQ-5D			
Median (Q1 and Q3)	0.80 (0.61 and 1.00)	0.76 (0.69 and 1.00)	0.80 (0.62 and 1.00)
COMI difference			
Median (Q1 and Q3)	-5.00 (-7.00 and -2.75)	-4.50 (-6.00 and -3.00)	-4.80 (-6.71 and -3.00)
ODI difference			
Median (Q1 and Q3)	-21.12 (-37.50 and -10.00)	-28.33 (-43.50 and -13.00)	-25.00 (-42.00 and -10.50)
EQ-5D difference			
Median (Q1 and Q3)	0.26 (0.09 and 0.42)	0.33 (0.16 and 0.67)	0.31 (0.13 and 0.54)

## Discussion

The gold standard in the treatment of lumbar disc herniation continues to be conventional microdiscectomy. The classic surgical approach, however, raises issues related to the extent of muscle retraction and dissection, bone resection, and the size of the surgical wound. Minimally invasive techniques, such as TMD and PELD, have been associated with good results, allowing the preservation of the paraspinal muscles, decreased postoperative pain, and early discharge [9].

Open interlaminar access has been described since the early 20th century; the application of microscope-assisted surgery and the advent of TMD marked a significant development, offering patients a less invasive option without compromising surgical efficacy. Endoscopic-assisted procedures were reported in the late 1990s, optimizing the route to the spinal canal under continuous visualization [6]; technological advances in optical quality led to a paradigm shift and the growing use of endoscopy in recent years.

In our study, at 12-month follow-up, PELD resulted in similar outcomes compared to TMD for the treatment of disc herniation, in a PSM sample. This equivalence was observed for COMI, ODI, and EQ-5D, as well as for days to return to work and surgical complications. The duration of postoperative pain medication was lower in the PELD group.

Despite its increasing use, PELD is a technology with a demanding learning curve. A surgeon with experience in minimally invasive surgery (MIS) may have doubts about switching to an endoscopic approach. This learning curve may lead to an initial increase in complications, such as higher rates of nerve root and dural injuries. On the other hand, tubular retractors can be associated with a reduced working space and field of view leading to incomplete decompression and higher recurrence rates [10]. Our study supports the efficacy and safety of PELD, when compared to tubular discectomy.

Similar clinical improvements at one-year follow-up or longer have been reported in comparison studies between minimally invasive discectomy and conventional approaches [11,12]. The advantage of minimally invasive spine surgical techniques is well-documented in the early postoperative period, leading to a faster recovery [13,14].

In a review of aggregated data from 11 studies comparing open discectomy, TMD, and PELD, Rasouli et al. [15] reported that minimally invasive techniques were associated with worse leg pain than open discectomy at follow‐up ranging from six months to two years; however, those differences were small and not clinically meaningful. The adverse events are similar in both groups, but they were rare, limiting the evidence related to complications. Among the patients we examined, five individuals (4.6%) experienced surgery-related complications. Specifically, three cases occurred in the PELD group, while the remaining two cases were observed in the TMD group.

MIS techniques have been associated with slightly higher rehospitalization rates due to recurrent disc herniation; however, surgical re-intervention rates seem to be similar between MIS and open techniques [10]. In our study, four patients underwent reoperation for recurrent disc herniation, two in each group.

There are few reports available in the literature comparing PELD to TMD. To our best knowledge, only two studies have done specifically this comparison. Yoon et al. [9] compared 25 patients submitted to PELD with 26 patients submitted to TMD with a follow-up of six months. The outcomes were comparable between the groups, and the authors concluded that PELD may be performed safely and effectively. In another study, Porto et al. [10] compared 71 patients submitted to PELD with 38 patients submitted to TMD, but this work only included single-level foraminal nerve root compressions. Clinical outcomes between procedures were similar, and severe foraminal stenosis was correlated to poorer outcomes; however, this correlation was stronger in the PELD group.

Given the observational nature of the current study, the matched methodology allowed a more robust comparison, as proven by the better balance of the chosen covariates, improving the validity of this work. However, further studies with randomization are recommended to clarify this question and support our results.

The limitations of this study include the lack of PROMs in the early postoperative period, which could have allowed the recognition of differences in the early outcomes. On the other hand, a limited follow-up of one year can lead to an underestimation of new pain episodes and reoperation rates. Another source of bias can be the absence of covariates related to preoperative imaging.

## Conclusions

The similar outcomes observed between PELD and TMD in our study suggest that both procedures are effective in treating lumbar disc herniation.

PELD represents an innovative and minimally invasive option for individuals suffering from symptomatic lumbar disc herniations, serving as an alternative to microdiscectomy. Based on comparisons with existing literature, PELD emerges as a safe and effective surgical procedure for addressing lumbar disc herniation. This technique has now become well-established, offering advantages such as minimal manipulation of the skin and soft tissues, enhanced intraoperative visualization with magnified images, and favorable cosmetic results. In our study, comparable outcomes were observed one year after surgery using PROMs related to pain, disability, and quality of life.

## References

[1] Gadjradj PS, Arts MP, van Tulder MW, Rietdijk WJ, Peul WC, Harhangi BS (2017). Management of symptomatic lumbar disk herniation: an international perspective. Spine (Phila Pa 1976).

[2] Kerr D, Zhao W, Lurie JD (2015). What are long-term predictors of outcomes for lumbar disc herniation? A randomized and observational study. Clin Orthop Relat Res.

[3] Clark AJ, Safaee MM, Khan NR, Brown MT, Foley KT (2017). Tubular microdiscectomy: techniques, complication avoidance, and review of the literature. Neurosurg Focus.

[4] Muthu S, Ramakrishnan E, Chellamuthu G (2021). Is endoscopic discectomy the next gold standard in the management of lumbar disc disease? Systematic review and superiority analysis. Global Spine J.

[5] Kogias E, Vougioukas VI, Hubbe U, Halatsch ME (2007). Minimally invasive approach for the treatment of lateral lumbar disc herniations. Technique and results. Minim Invasive Neurosurg.

[6] Ruetten S, Komp M, Merk H, Godolias G (2008). Full-endoscopic interlaminar and transforaminal lumbar discectomy versus conventional microsurgical technique: a prospective, randomized, controlled study. Spine (Phila Pa 1976).

[7] Damasceno LH, Rocha PA, Barbosa ES, Barros CA, Canto FT, Defino HL, Mannion AF (2012). Cross-cultural adaptation and assessment of the reliability and validity of the Core Outcome Measures Index (COMI) for the Brazilian-Portuguese language. Eur Spine J.

[8] Mannion AF, Elfering A, Staerkle R (2005). Outcome assessment in low back pain: how low can you go?. Eur Spine J.

[9] Yoon SM, Ahn SS, Kim KH, Kim YD, Cho JH, Kim DH (2012). Comparative study of the outcomes of percutaneous endoscopic lumbar discectomy and microscopic lumbar discectomy using the tubular retractor system based on the VAS, ODI, and SF-36. Korean J Spine.

[10] Porto GB, Cisewski SE, Wolgamott L, Frankel BM (2021). Clinical outcomes for patients with lateral lumbar radiculopathy treated by percutaneous endoscopic transforaminal discectomy versus tubular microdiscectomy: a retrospective review. Clin Neurol Neurosurg.

[11] Ruan W, Feng F, Liu Z, Xie J, Cai L, Ping A (2016). Comparison of percutaneous endoscopic lumbar discectomy versus open lumbar microdiscectomy for lumbar disc herniation: a meta-analysis. Int J Surg.

[12] Kim SK, Kang SS, Hong YH, Park SW, Lee SC (2018). Clinical comparison of unilateral biportal endoscopic technique versus open microdiscectomy for single-level lumbar discectomy: a multicenter, retrospective analysis. J Orthop Surg Res.

[13] Park SM, Kim GU, Kim HJ, Choi JH, Chang BS, Lee CK, Yeom JS (2019). Is the use of a unilateral biportal endoscopic approach associated with rapid recovery after lumbar decompressive laminectomy? A preliminary analysis of a prospective randomized controlled trial. World Neurosurg.

[14] Qin F, Zhang Z, Zhang C, Feng Y, Zhang S (2020). Effect of time to first ambulation on recurrence after PELD. J Orthop Surg Res.

[15] Rasouli MR, Rahimi-Movaghar V, Shokraneh F, Moradi-Lakeh M, Chou R (2014). Minimally invasive discectomy versus microdiscectomy/open discectomy for symptomatic lumbar disc herniation. Cochrane Database Syst Rev.

